# Immunotherapy Resistance in dMMR/MSI‐H Colorectal Cancer: Unraveling Mechanisms and Exploring Overcoming Strategies

**DOI:** 10.1155/jimr/5084171

**Published:** 2026-08-12

**Authors:** Ke Zhou, Ping Lu, Hongli Xu, Xinjun Liang

**Affiliations:** ^1^ Department of Abdominal Oncology, Hubei Cancer Hospital, Tongji Medical College, Huazhong University of Science and Technology, Wuhan 430079, Hubei, China, hust.edu.cn; ^2^ Hubei Provincial Clinical Research Center for Colorectal Cancer, Wuhan 430079, Hubei, China; ^3^ Wuhan Clinical Research Center for Colorectal Cancer, Wuhan 430079, Hubei, China

**Keywords:** DNA mismatch repair deficient (dMMR), drug resistance, immune checkpoint inhibitors (ICIs), metastatic colorectal cancer (mCRC), microsatellite instability-high (MSI-H)

## Abstract

Colorectal cancer (CRC) remains a leading cause of cancer‐related mortality worldwide. Approximately 15% of localized and 5% of metastatic cases exhibit mismatch repair deficiency (dMMR) or high microsatellite instability (MSI‐H). While immune checkpoint inhibitors (ICIs) have revolutionized the first‐line treatment for this subgroup, 15%–46% of patients experience primary resistance, and a subset of responders eventually acquires resistance. This review synthesizes the multifaceted mechanisms underlying ICI resistance in dMMR/MSI‐H CRC. We delineate tumor‐intrinsic alterations, including defects in the antigen presentation machinery (specifically transporter associated with antigen processing [TAP]1/TAP2 and β_2_‐microglobulin [β_2_m]), oncogenic signaling via the Wnt/β‐catenin and JAK/STAT pathways, and epigenetic remodeling involving ARID1A. Furthermore, we explore the role of the immunosuppressive tumor microenvironment (TME), characterized by T‐cell exclusion and myeloid‐derived suppressor cell (MDSC) accumulation. To address these barriers, we evaluate the clinical potential of third‐generation ICIs targeting lymphocyte activation gene 3 (LAG‐3), T‐cell immunoglobulin and mucin‐domain‐containing‐3 (TIM‐3), and TIGIT, as well as emerging biomarker strategies such as gut microbiome modulation and circulating tumor DNA (ctDNA) dynamics. By integrating these mechanistic insights with novel therapeutic approaches, including bispecific antibodies (BsAbs) and adoptive cell transfer, this review aims to provide a roadmap for overcoming resistance and advancing precision immunotherapy in dMMR/MSI‐H CRC.

## 1. Introduction

Colorectal cancer (CRC) is a leading cause of cancer‐related mortality globally. Epidemiological data from 2022 indicate that CRC ranks third in incidence and second in mortality worldwide, underscoring its substantial public health burden [1]. Approximately 15% of localized CRC cases exhibit mismatch repair deficiency (dMMR); however, this proportion declines with the advancing disease stage [2]. In metastatic CRC (mCRC), only about 5% of cases are dMMR with high microsatellite instability (MSI‐H), while the vast majority (95%) are microsatellite stable (MSS) [3].

In contrast to MSS tumors, dMMR/MSI‐H mCRC is characterized by a high tumor mutational burden (TMB) and abundant neoantigen production, rendering it particularly susceptible to immune checkpoint inhibitors (ICIs) [4, 5]. ICIs have thus become the standard first‐line therapy for this subgroup. However, significant clinical challenges remain: approximately 15%–46% of patients exhibit primary resistance, and 5%–25% of initial responders eventually develop acquired resistance [6]. The underlying mechanisms driving this resistance are complex and have not been fully elucidated.

Current research often focuses on isolated pathways, lacking a comprehensive synthesis of the interplay between tumor‐intrinsic alterations and the dynamic tumor immune microenvironment. Furthermore, distinct biological features of CRC, such as the frequent involvement of the Wnt/β‐catenin pathway and Lynch syndrome‐related genetics, necessitate a CRC‐specific resistance framework. This review aims to bridge this gap by systematically summarizing recent advances in resistance mechanisms, highlighting the crosstalk among antigen presentation defects, signaling pathways, and the tumor microenvironment (TME), and exploring novel strategies to overcome resistance in dMMR/MSI‐H mCRC.

## 2. Current Landscape of Immunotherapy in dMMR/MSI‐H mCRC

### 2.1. Standard‐of‐Care (SOC) ICIs

The introduction of ICIs has fundamentally altered the treatment paradigm for dMMR/MSI‐H mCRC. Based on pivotal trials such as KEYNOTE‐177 and CheckMate‐8HW, anti‐programed death‐1 (PD‐1) monotherapy (e.g., pembrolizumab) or combination therapy (nivolumab + ipilimumab) has become the established first‐line SOC for this patient population. Unlike the heterogeneous responses observed in other tumor types, dMMR/MSI‐H CRC demonstrates profound and durable responses to the ICI blockade. The 2025 updated analysis of KEYNOTE‐177 confirmed that pembrolizumab significantly prolongs progression‐free survival (PFS) compared to chemotherapy (16.5 vs. 8.2 months) [7]. Similarly, the phase III CheckMate‐8HW trial demonstrated a remarkable 2‐year PFS rate of 72% with the nivolumab plus ipilimumab regimen, underscoring the potency of dual checkpoint blockade in CRC [8]. These regimens not only offer superior long‐term disease control but also exhibit a more favorable safety profile compared to traditional cytotoxic therapies. The strong clinical results observed in these trials have established dMMR/MSI‐H CRC as a model for effective immunotherapy in solid tumors. (Table 1)

**Table 1:** The clinical studies of ICIs in dMMR/MSI‐H colorectal cancer.

NCT number	Interventions	Enrollment	Primary endpoint	Outcome
NCT02460198 (KEYNOTE‐164)	Pembrolizumab	124 dMMR/MSI‐H mCRC participants with ≥1 prior line of therapy [9].	ORR	≥2 prior lines: ORR = 33% ≥1 prior lines: ORR = 33%
NCT02563002 (KEYNOTE‐177)	Pembrolizumab vs. chemotherapy	307 untreated dMMR/MSI‐H mCRC participants [7].	PFS; OS	Pembrolizumab: PFS = 16.5 months chemotherapy: PFS = 8.2 months (follow‐up time: 32.4 months)
NCT03026140 (NICHE)	Ipilimumab + nivolumab	57 participants with resectable stage I–III colon adenocarcinoma (35 dMMR/MSI‐H, 22 pMMR/MSS) [10].	DCR	DCR = 86%
NCT04008030 (CheckMate‐8HW)	Nivolumab + ipilimumab vs. chemotherapy	303 treatment‐naïve dMMR/MSI‐H mCRC participants (2:1 randomization) [8].	PFS	Nivolumab plus ipilimumab: PFS = 54.1 months chemotherapy: PFS = 5.9 months
	Nivolumab + ipilimumab vs. nivolumab vs. chemotherapy	707 immunotherapy‐naïve dMMR/MSI‐H mCRC participants (2:2:1 randomization) [8].		Nivolumab plus ipilimumab: PFS was not reached nivolumab: PFS = 39.3 months
NCT02060188 (CheckMate 142)	Nivolumab + low‐dose ipilimumab	119 dMMR/MSI‐H mCRC participants pretreated with fluoropyrimidines, oxaliplatin, or irinotecan [11].	ORR	ORR = 69% DCR = 84%
	Nivolumab + ipilimumab			ORR = 58% DCR = 81%
	Nivolumab			ORR = 39% DCR = 69%
NCT03150706	Avelumab	33 dMMR/MSI/POLE‐mutated participants [12].	ORR	ORR = 24.2%
NCT03186326 (SAMCO‐PRODIGE 54)	Avelumab	132 participants (RAS‐mutant: 45%, BRAF V600E: 15%, RAS/BRAF WT: 40%) [13].	PFS	PFS = 12 months
NCT02715284 (GARNET)	Dostarlimab	363 dMMR/MSI‐H/POLE‐altered tumors (105 dMMR CRC) [14].	ORR	Colorectal cancer ORR = 43.5%

Abbreviations: DCR, disease control rate; ORR, overall response rate.

### 2.2. Third‐Generation ICIs

Third‐generation ICIs target immune regulatory pathways beyond cytotoxic T‐lymphocyte‐associated protein 4 (CTLA‐4) and PD‐1/programed death‐ligand 1 (PD‐L1), primarily including lymphocyte‐activation gene 3 (LAG‐3), T‐cell immunoglobulin and mucin‐domain‐containing‐3 (TIM‐3), and T‐cell immunoreceptor with immunoglobulin and immunoreceptor tyrosine‐based inhibition motif (ITIM) domains (TIGIT). Unlike first‑generation and second‑generation inhibitors, third‐generation agents frequently employ bispecific antibodies (BsAbs) or combination therapies to overcome resistance.

In dMMR CRC, LAG‐3 is highly expressed on tumor‐infiltrating lymphocytes (TILs). The NICHE‐3 trial demonstrated that neoadjuvant nivolumab plus relatlimab achieved 68% pathological complete response (pCR) and 92% major pathological response (mPR) in locally advanced dMMR colon cancer [15]. TIM‐3 expression is associated with worse outcomes in CRC. In the CALGB/SWOG 80405 trial, high TIM‐3 expression correlated with shorter survival and appears to be involved in EGFR resistance pathways [16]. In a CT26 mouse model, dual PD‑1/TIM‑3 blockade inhibited tumor progression and reduced T‑cell exhaustion [17]. TIGIT is upregulated on CD4^+^, CD8^+^, NK, and γδ T cells in CRC, and TIGIT^+^ CD8^+^ T cells show reduced cytokine production [18]. In MSS CRC, cancer‐associated fibroblasts (CAFs) suppress Vδ1^+^ γδ T cells via the TIGIT‐NECTIN2 axis, an effect reversed by the TIGIT blockade [19]. The NEOTERIC trial demonstrated that neoadjuvant chemoradiotherapy followed by atezolizumab plus tiragolumab increased pCR rates in locally advanced rectal cancer [20].

**Figure 1:**
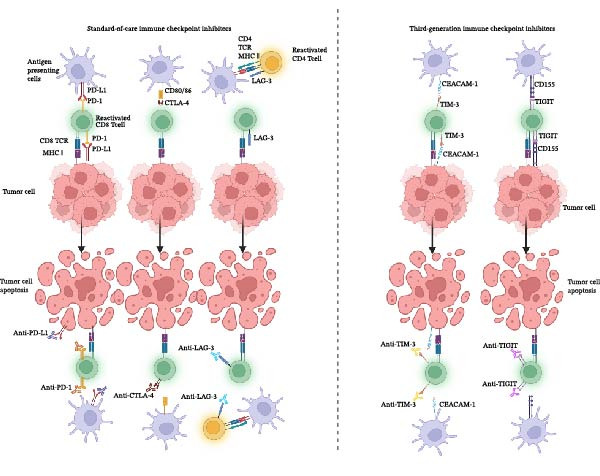
Comparative mechanisms of standard and next‐generation immune checkpoint blockade.

Collectively, these targets cover distinct CRC subtypes—dMMR (LAG‑3 and TIM‑3) and MSS (TIGIT)—and diverse immune cell populations, justifying continued clinical investigation (Figure 1).

This schematic depicts how ICIs reverse immunosuppressive signaling to promote tumor cell apoptosis. The left panel highlights standard therapies targeting the PD‐1/PD‐L1, CTLA‐4, and LAG‐3 axes to reactivate CD8^+^ and CD4^+^ T cells. The right panel illustrates third‐generation strategies targeting TIM‐3 (via CEACAM‐1) and TIGIT (via CD155), which aim to overcome resistance by blocking alternative inhibitory pathways on TILs.

## 3. Mechanisms of Immunotherapy Resistance in dMMR/MSI‐H mCRC

**Figure 2:**
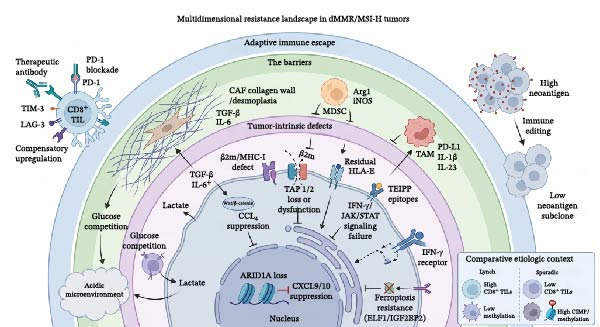
Multidimensional resistance landscape in dMMR/MSI‐H tumors. Resistance arises from the interplay between tumor‐intrinsic alterations and the immunosuppressive microenvironment (TME). (A) Defective antigen presentation: mutations in β_2_‐microglobulin (β_2_m) or downregulation of TAP1/2 allow evasion of CD8^+^ T‐cell recognition. β_2_m loss ablates classical MHC‐I, while TAP deficiency promotes alternative antigen presentation. (B) Oncogenic signaling and epigenetics: cytoplasmic Wnt/β‐catenin activation suppresses CCL4, impairing dendritic cell recruitment. Concurrent nuclear loss of AT‐rich interaction domain 1A (ARID1A) represses T‐cell chemokines (e.g., CXCL9/10), limiting infiltration. (C) Metabolic adaptation: an ELF1/IGF2BP2/FAM111B cascade confers ferroptosis resistance. High glycolytic activity depletes glucose and accumulates lactate, suppressing effector T‐cell function. (D) Adaptive resistance: tumors upregulate compensatory checkpoints (TIM‐3 and LAG‐3) on exhausted T cells, driving resistance to single‐agent therapy.

Resistance to ICIs in dMMR/MSI‐H CRC is not driven by a single factor but arises from a dynamic interplay between tumor‐intrinsic alterations and the host immune microenvironment. While some mechanisms confer primary resistance, others emerge as adaptive responses under therapeutic pressure. Here, we dissect these mechanisms through a CRC‐specific lens (Figure 2).

### 3.1. Defects in Antigen Presentation Machinery

The efficacy of ICIs fundamentally depends on the recognition of tumor neoantigens by CD8^+^ T cells via MHC class I molecules. The disruption of this axis represents the most well‐characterized resistance mechanism in MSI‐H CRC. Although MSI‐H tumors typically generate abundant neoantigens [21], approximately 17% of dMMR/MSI‐H tumors exhibit low TMB, which correlates with decreased TIL infiltration and reduced PD‐L1 levels, suggesting an increased likelihood of immunotherapy failure [22–24]. Beyond this quantitative defect, dysfunction in the MHC I antigen processing machinery (APM) directly impairs immune recognition [25].

#### 3.1.1. Transporter Associated With Antigen Processing (TAP) Deficiency

TAP is a heterodimeric complex (TAP1/TAP2) that translocates proteasomal peptides into the endoplasmic reticulum for MHC I loading [26]. TAP defects lead to MHC I downregulation and subsequent immune evasion. In CRC, TAP1 downregulation correlates with reduced immune cell infiltration and is an independent poor prognostic factor in stage I–II disease, partly due to promoter hypermethylation [27]. CRC exhibits two distinct molecular pathways of MHC I loss: β_2_‑microglobulin (β_2_m) inactivation in MSI‑positive tumors and LMP7/TAP2 downregulation in MSI‑negative tumors [28]. This subtype‑specific defect pattern helps explain ICI resistance even in dMMR/MSI‑H CRC. In mouse models, TAP1‑deficient tumors resist anti‑PD‑1 therapy, but combined Qa‑1 b blockade partially reverses this resistance [29]. Notably, TAP‐defective CRC cells present an alternative repertoire of T‐cell epitopes termed T‐cell epitopes associated with impaired peptide processing (TEIPP), which are derived from non‐mutant self‐proteins and represent a potential vulnerability for immunotherapeutic targeting in TAP‐deficient CRC.

#### 3.1.2. β_2_m Deficiency

The MHC class I/HLA complex is structurally characterized by α‐chains noncovalently associated with β_2_m. In MSI‐H CRC, β_2_m alterations occur in approximately 24%–40% of cases due to the repetitive nature of the β_2_m coding sequence, frequently correlating with the loss of protein expression [30]. One case report described a CRC harboring a β_2_m gene chimera that became resistant to ICI therapy, underscoring its role as an emerging predictive biomarker [31]. Of note, in contrast to melanoma, where β_2_m loss leads to complete HLA‐I absence, CRC with β_2_m inactivation often retains residual HLA‐E expression, which may partially engage NK cells and compensate for HLA‐I loss. Furthermore, evidence from murine models indicates that CD4^+^ T lymphocytes can orchestrate tumor rejection independent of β_2_m, suggesting that the ICI response in dMMR CRC may engage distinct immunological pathways compared with those in melanoma and lung cancers [32].

### 3.2. Oncogenic Signaling Pathways Driving Immune Exclusion

#### 3.2.1. Wnt/β‐Catenin Activation

Aberrant Wnt/β‐catenin activation drives immune evasion and ICI resistance in CRC. In CRC patients, β‐catenin hyperactivation correlates with reduced CD8^+^ T‐cell infiltration [33]. Mechanistically, β‐catenin suppresses CCL4 expression, impairing CD103^+^ dendritic cell recruitment and subsequent CD8^+^ T‐cell activation [33]. A recent study identified the USP21‐β‐catenin‐ATF3‐CCL4 axis as a key regulator of T‐cell exclusion in CRC; targeting USP21 with BAY‐805 synergized with anti‐PD‐1 therapy in both MSI‐H and MSS CRC models [34]. Additionally, β‐catenin activation drives γδ T‐cell exclusion by suppressing BTNL gene expression in colon cancer [35]. Therapeutic β‐catenin inhibition (iCRT14) in CRC mouse models upregulated T‐cell‑recruiting chemokines (CXCL9/10/11) and synergized with tumor vaccines [36]. These findings establish Wnt/β‐catenin as a central mediator of ICI resistance in CRC, warranting combination strategies targeting this pathway.

#### 3.2.2. JAK/STAT and PI3K/AKT Alterations

The JAK/STAT signaling pathway is often dysregulated in CRC. Mutations in JAK1/JAK2 disrupt interferon‐gamma (IFN‐γ) signaling, rendering tumor cells insensitive to T‐cell‐derived cytotoxicity even when infiltrated [37, 38]. Concurrently, PI3K/AKT activation in CRC, frequently driven by PTEN loss, promotes PD‐L1 upregulation and metabolic competition, thereby reinforcing immune evasion and impairing T‐cell‐mediated antitumor immunity [39–43]. PTEN loss also contributes to an immunosuppressive TME by promoting the recruitment of myeloid‐derived suppressor cells (MDSCs) and regulatory T cells (Tregs), as well as by reducing CD8^+^ T cell infiltration and cytotoxicity [44]. Importantly, these pathways often co‐occur with Wnt activation, creating a multilayered barrier to immune infiltration and ICI responses. Consequently, despite elevated PD‐L1 expression, PTEN deficiency has been shown to predict poor treatment responses in patients receiving immunotherapy as the net effect of these coordinated immunosuppressive mechanisms outweighs the potential benefit of a single‐axis PD‐1/PD‐L1 blockade [45].

#### 3.2.3. Epigenetic Remodeling: ARID1A and the SWI/SNF Complex

A critical distinction of CRC is the high prevalence of ARID1A mutations, occurring in approximately 15%–20% of cases. ARID1A encodes a core subunit of the SWItch/sucrose nonfermentable (SWI/SNF) chromatin remodeling complex [46, 47]. Unlike structural APM defects, ARID1A loss does not directly impair peptide processing or MHC‐I loading. Instead, it can epigenetically repress the expression of key chemokines (e.g., CXCL9/10) that are critical for T‐cell recruitment [48]. This may contribute to an immune‐excluded TME in specific subsets, although the overall immunologic role of ARID1A deficiency in CRC is complex and can also be associated with immune activation in certain contexts [49]. This epigenetic dysregulation links CRC‐specific chromatin remodeling directly to immune evasion and ICI resistance, representing an emerging therapeutic target [47].

### 3.3. Inhibition of the TME

The TME comprises diverse cellular and acellular components that collectively shape ICI responsiveness [50]. MDSCs accumulate in the TME, where they impair T‐ and NK‐cell activity through arginase‐1 and iNOS secretion [51, 52]. CAFs in CRC form dense desmoplastic barriers that physically exclude T cells while secreting transforming growth factor‐beta (TGF‐β) and interleukin‐6 (IL‐6) [53]. In CRC models, CD70‐expressing CAFs attract Tregs to tumor sites [54]. Notably, TGF‐β signaling in CRC CAFs synergizes with Wnt/β‐catenin to reinforce the immune‐excluded phenotype.

In addition to cellular components, soluble factors and metabolic byproducts also contribute to the immunosuppressive TME. Emerging research has demonstrated that inflammatory mediators within the TME can upregulate PD‐L1 levels, potentially modulating the response to PD‐1/PD‐L1 blockade therapy [55]. TAMs secrete various protumorigenic cytokines (including interleukin‐1β [IL‐1β], IL‐6, and interleukin‐23 [IL‐23]) that facilitate cancer development in colorectal carcinoma and other malignancies [56]. Notably, these TAMs also overexpress immunosuppressive ligands such as PD‐L1/PD‐L2, directly mediating the functional suppression of T cells. In addition, the accumulation of catabolic waste products has been shown to diminish ICI effectiveness. Therapeutic interventions targeting these processes, such as enhanced ammonia clearance in mCRC patients, have demonstrated the potential to restore antitumor immune responses [57].

Additionally, Lynch syndrome‐associated dMMR tumors typically exhibit higher CD8^+^ T‐cell infiltration and lower methylation burden compared to sporadic CpG island methylator phenotype (CIMP)‐high dMMR CRCs [58, 59]. This suggests that sporadic dMMR CRC may harbor additional epigenetic immune silencing mechanisms beyond MMR deficiency alone [60], potentially explaining differential ICI responses between these two etiologies [61].

### 3.4. Adaptive Resistance and T Cell Exhaustion

Prolonged antigenic stimulation drives T cells toward exhaustion, a state characterized by elevated inhibitory marker expression (PD‐1 and CTLA‐4) and diminished effector function [62, 63]. While PD‐1 blockade can reinvigorate exhausted T cells, terminally exhausted CD8^+^ TILs no longer respond to the PD‐1/PD‐L1 blockade [64, 65]. In CRC, exhausted T cells persisting after PD‐1 blockade upregulate alternative inhibitory receptors. TIM‐3 and LAG‐3 are elevated on residual TILs post‐treatment, marking a transition toward terminal exhaustion [66]. This compensatory circuit provides the biological rationale for dual blockade strategies currently being validated in CRC trials.

Metabolic competition and clonal evolution further drive acquired resistance. Tumor cells rewire their energy metabolism toward aerobic glycolysis, increasing lactate production and creating an acidic TME that impairs T‐cell function [67]. Inflammation and metabolic reprograming are intimately linked in this process. Tumor‐derived inflammatory mediators, including IL‐6, TNF‐α, and TGF‐β, activate metabolic checkpoints such as hypoxia‐inducible factor 1‐alpha (HIF‐1α) and MYC proto‐oncogene (c‐Myc), which in turn drive the Warburg effect and lactate accumulation [68]. This metabolic‐inflammatory crosstalk creates a vicious cycle in which inflammation promotes metabolic adaptation that further fuels inflammatory signaling.

Within this metabolically hostile environment, T cells undergo profound functional changes. CD8^+^ T cells exhibit progressive exhaustion, characterized by reduced IFN‐γ secretion, impaired cytotoxic function, and upregulation of multiple inhibitory receptors, including PD‐1, TIM‐3, and LAG‐3 [69]. Concurrently, the abundance of Tregs is significantly increased in the TME, elevating the Treg/CD8^+^ T‐cell ratio and thereby suppressing antitumor immunity. Mechanistically, lactate and other metabolic byproducts skew T‐cell differentiation toward immunosuppressive phenotypes, favoring Treg and T helper 2 (Th2) subsets while restricting antitumor T helper 1 (Th1) and T helper 17 (Th17) responses [70, 71]. This metabolism‐driven shift in the T‐cell landscape represents a key barrier to effective immunotherapy.

Epigenetic dysregulation also contributes to metabolic adaptation; the ELF1/IGF2BP2/FAM111B cascade has been shown to drive CRC progression and confer resistance to ferroptosis, a form of iron‐dependent cell death [72]. Metabolic adaptations involving mTOR and AMPK pathways can also influence treatment resistance [73]. Longitudinal analyses reveal that ICI treatment selects for subclones with reduced neoantigen burdens or enhanced survival pathways—a process accelerated by the genomic instability inherent to MSI‐H tumors, enabling rapid adaptation to immune pressure.

## 4. Future Perspectives and Biomarker Development

Overcoming resistance to ICIs in dMMR/MSI‐H CRC requires robust biomarkers to guide precision immunotherapy and novel strategies to circumvent resistance. Here, we categorize emerging biomarkers by their level of clinical validation and discuss the corresponding therapeutic approaches. To provide a comprehensive overview of the current landscape, Table 2 summarizes these biomarkers alongside their representative evidence sources and key translational limitations.

**Table 2:** Stratification of biomarkers for immunotherapy in dMMR/MSI‐H colorectal cancer: clinical validation, evidence sources, and limitations.

Category	Biomarker/strategy	Key evidence	Clinical utility	Key limitations
Clinically established	dMMR/MSI status	KEYNOTE‐177; CheckMate‐8HW	First‐line selection	~17% TMB‐low subset
	TMB (continuous)	Post hoc analyses; ATOMIC consortium	Prognostic stratification	CRC‐specific cutoffs pending
	*POLE/POLD1* mutations	NCT05385480 (Phase II)	Identifies ultramutators	Benefit restricted to subtypes
	Immunoscore (CD3^+^/CD8^+^)	Large‐scale CRC cohorts; AtezoTRIBE	Superior to TNM staging	Spatial metrics unstandardized
	PD‐L1 (CPS/TPS)	Multicancer Phase II/III trials	Limited in MSI‐H	Weak predictive value
Translational validation	Microbiome (*A. muciniphila*, *F. nucleatum*)	CRC cohorts and translational studies	Noninvasive stratification	Causality unconfirmed
	ctDNA dynamics (MRD)	NICHE‐2 (Phase II)	Neoadjuvant surrogate endpoint	Prospective validation needed
	*ARID1A*/CIMP‐high	Retrospective CRC cohorts	Identifies immune‐excluded	Lacks prospective data
	Transcriptomic signatures	IMMETPRED; 8‐gene signature	Guides combination therapy	Assay standardization lacking
Exploratory	Circulating CD8^+^CD28^+^ T cells	Retrospective mCRC cohort	Dynamic monitoring	CRC validation absent
	PD‐L1^+^ CTCs/exosomal miRNAs	NSCLC‐predominant data	Exploratory	CRC data lacking
	Nanomedicines/MDSC‐targeting	Murine CRC models	Preclinical stage	No human CRC efficacy data

### 4.1. Clinically Established Biomarkers

dMMR/MSI status remains the primary selection criterion for ICI therapy in CRC. Within dMMR tumors, TMB quantification provides additional prognostic granularity: retrospective analyses of KEYNOTE‐177 and CheckMate‐8HW have demonstrated that higher TMB correlates with improved PFS and OS, independent of the MSI status. The ATOMIC consortium has established standardized TMB thresholds for CRC, moving toward harmonized cutoffs that account for CRC‐specific mutational signatures.

Beyond core MMR/TMB metrics, alterations in the exonuclease domains of POLE and POLD1 confer ultramutator phenotypes and have been associated with enhanced ICI sensitivity. However, a phase II study (NCT05385480) demonstrated significant heterogeneity among POLE/POLD1 mutations, indicating that not all such mutations are universal indicators of immunotherapy benefit. Only those harboring exonuclease domain alterations, concurrent PBRM1 loss, or MSI‐high status achieve meaningful responses, necessitating further biomarker segmentation [74].

T‐cell infiltration and spatial organization reflect the actual immune interface and offer complementary information to genomic metrics. The Immunoscore quantifies CD3^+^ and CD8^+^ T cells at the tumor core and invasive margin and has been extensively validated in CRC as a predictor of ICI response that outperforms TNM staging [75]. Recent extensions incorporating spatial metrics, such as CD8^+^ proximity to tumor cells and tertiary lymphoid structure maturity, further refine prediction. These approaches are particularly useful for identifying “immune‐excluded” dMMR tumors that may benefit from combination therapies targeting Wnt or TGF‐β pathways.

PD‐L1 expression has been evaluated as a predictive biomarker in CRC [76], but its utility remains limited by assay variability, temporal heterogeneity, and suboptimal performance in MSI‐H populations. These limitations indicate that no single tissue‐based marker captures the full complexity of ICI response and reinforce the value of complementary approaches discussed in subsequent sections [77].

### 4.2. Emerging Biomarkers in Translational Validation

The gut microbiome modulates the ICI response in CRC through direct anatomical proximity and local immune crosstalk. *Akkermansia muciniphila* (*A. muciniphila*) enhances CD8^+^ T‑cell infiltration and function within the TME, thereby potentiating PD‑1/PD‑L1 blockade [78, 79]. In contrast, enrichment of *Fusobacterium nucleatum* (*F. nucleatum*) promotes resistance via TLR4/MYD88‑mediated immune suppression and PD‑L1 upregulation [80]. Emerging evidence highlights that gut microbial metabolites exert crucial regulatory effects within the tumor immune microenvironment, playing dual roles in either promoting or suppressing antitumor immunity [81]. Multiple ongoing trials are testing microbiome modulation strategies, including fecal microbiota transplantation and probiotic supplementation, in combination with ICIs in CRC.

Circulating tumor DNA (ctDNA) dynamics have emerged as a promising surrogate endpoint for treatment monitoring. In the neoadjuvant setting, the NICHE‑2 trials demonstrated that undetectable post‑treatment ctDNA strongly predicts pCR and long‑term recurrence‑free survival in dMMR colon cancer treated with dual ICI blockade [82]. This positions ctDNA as a potential surrogate endpoint for accelerating trial design and informing adjuvant therapy decisions. In metastatic NSCLC, early ctDNA reduction correlates with higher response rates and prolonged progression‑free survival, whereas subsequent ctDNA increases precede radiographic progression [83].

Epigenetic biomarkers, including ARID1A mutations and CIMP‑high status, define immunologically distinct subsets within dMMR CRC and may identify patients refractory to ICIs despite MSI‑H status [84, 85]. Transcriptomic signatures integrating IFN‑γ signaling and T‑cell exhaustion have similarly shown predictive value in retrospective CRC analyses, though standardized assays and prospective validation are required for clinical adoption.

### 4.3. Therapeutic Strategies Informed by Biomarker Stratification

Biomarker‐based stratification directly informs therapeutic decision‐making. Dual PD‐1/CTLA‐4 blockade with nivolumab plus ipilimumab is now a first‐line standard for dMMR CRC, validated by CheckMate‐142 and CheckMate‐8HW. Emerging pairings, including PD‐1/LAG‐3 and PD‐L1/TIGIT, are under investigation to improve responses while limiting toxicity.

In biomarker‐defined subsets, BRAF V600E‐mutant dMMR CRC may benefit from BRAF/EGFR inhibition combined with PD‐1 blockade. The NCT05217446 trial is evaluating encorafenib, cetuximab, and pembrolizumab in BRAF V600E‐mutant mCRC patients who have progressed on prior therapy. The BREAKWATER trial; however, reported grade ≥3 adverse events in 46% of participants, prompting consideration of optimized dosing or sequencing strategies [86].

Antiangiogenic agents combined with ICIs exert synergistic effects by reversing VEGF‐mediated immunosuppression. The REGONIVO trial reported a 33% ORR with regorafenib plus nivolumab in MSS‐type mCRC; the MSI‐H subgroup efficacy was not separately analyzed [87]. The ongoing ASTRUM‐015 trial (NCT05400122) is evaluating a PD‐1 blocker combined with bevacizumab and chemotherapy in MSS‐type CRC, with MSI‐H subgroup data pending.

Clinically developed BsAbs in CRC target a diverse range of pathways. Among immune checkpoint‐related pairs, PD‐1/CTLA‐4 and PD‐1/VEGF are the most frequently investigated combinations in CRC clinical trials. The PD‐1/CTLA‐4 BsAb, cadonilimab, has shown antitumor activity in locally advanced CRC, with multiple phase II trials demonstrating promising pathological response rates. The PD‐1/VEGF BsAb ivonescimab has also demonstrated encouraging activity in CRC, with ongoing phase II and III trials evaluating its efficacy in both first‐line and later‐line settings, including in anti‐PD‐1 refractory dMMR/MSI‐H CRC. Beyond bispecific formats, trispecific antibodies represent the next generation of engineered immunotherapies. IMT030122, a novel EpCAM/CD3/4‐1BB trispecific antibody, has demonstrated potent anti‐tumor activity in CRC mouse models by recruiting T cells and mediating EpCAM‐targeted T‐cell cytotoxicity [88].

In adoptive cell transfer, the CARAPIA‐1 trial evaluated GCC19CART in 10 patients with mCRC refractory to second‐line therapy; the 2 × 10^6^ CAR‐T‐cell/kg cohort (*n* = 5) achieved an 80% ORR, including one pCR [89]. Patient selection was based on GCC positivity by immunohistochemistry; comprehensive genomic profiling data are not reported.

## 5. Discussion and Conclusion

Resistance to immunotherapy in dMMR/MSI‐H CRC is driven by multidimensional and interconnected mechanisms, including antigen presentation defects, oncogenic pathway activation, epigenetic remodeling, and microenvironmental immunosuppression. While each mechanism has been individually characterized, their clinical relevance lies in how they co‐occur and interact within individual tumors to create multilayered barriers to ICI efficacy. Wnt/β‐catenin activation, JAK/STAT mutations, and MDSC accumulation often coexist, suggesting that single‐agent targeting is unlikely to overcome resistance and that combination strategies must be informed by the dominant resistance pathways in each patient.

A major advance in the field is the recognition that resistance mechanisms can be translated into predictive biomarker panels. Genomic markers, including MSI, TMB, POLE/POLD1, and ARID1A, provide information on tumor‐intrinsic immunogenicity. Spatial markers such as Immunoscore assess T‐cell infiltration and organization. Liquid biopsy markers including ctDNA and circulating CD8^+^CD28^+^ T cells [90] enable dynamic monitoring, while microbial markers reflect host‐gut immune crosstalk. The integration of these layers into unified models, potentially facilitated by machine learning, represents the frontier of precision immunotherapy. However, the value of such models must be demonstrated through improved patient outcomes and not technical performance alone.

Several critical gaps remain. First, prospective validation of emerging biomarkers in biomarker‐stratified trials is largely absent; most evidence remains retrospective or derived from exploratory cohort analyses. Second, the distinction between primary and acquired resistance is often blurred in cross‐sectional datasets, yet the two likely involve distinct biological processes and require different therapeutic strategies, including rechallenge, sequencing, or combination approaches, each requiring separate validation. Third, the translational gap between promising preclinical findings and clinical application remains wide. Although nanoparticle‐based strategies have demonstrated synergy with ICIs in murine CRC models [91], human data are lacking. Fourth, host factors such as germline immunogenetics and the gut microbiome have been underexplored as determinants of ICI resistance, yet they may significantly influence interpatient variability. These gaps underscore the need for adaptive trial designs with embedded biomarker discovery, standardized assay platforms, and collaborative consortia to pool real‐world data.

In conclusion, immunotherapy has transformed the treatment of dMMR/MSI‐H mCRC, yet primary and acquired resistance remains a substantial clinical challenge. Resistance arises from a dynamic interplay of tumor‐intrinsic alterations, including antigen presentation defects, oncogenic signaling pathways, and epigenetic remodeling, as well as extrinsic factors within the tumor immune microenvironment. The identification of robust predictive biomarkers, ranging from MSI/TMB and Immunoscore to ctDNA and microbiome signatures, is essential for guiding patient stratification and combination therapy selection. Novel therapeutic strategies, including third‐generation ICIs, BsAbs, and CAR‐T cells, are being actively developed to overcome resistance. Ultimately, a coordinated approach that integrates biomarker‐driven patient selection, rational combination strategies, and adaptive trial designs will be required to translate mechanistic insights into meaningful clinical benefits for patients with dMMR/MSI‐H CRC.

## Author Contributions

**Ke Zhou:** writing – original draft, visualization. **Ping Lu and Hongli Xu:** writing – review and editing, visualization. **Xinjun Liang:** conceptualization, investigation, methodology, writing – review and editing, supervision, project administration, funding Acquisition.

## Funding

This work was financially supported by the Project of the Joint Fund for Innovation and Development of the Natural Science Foundation of Hubei Province of China (Grant 2026AFC1224), Scientific Research Projects of Hubei Cancer Hospital (Grants 2024HBCHYN13 and 2025HBCHYN03), Talent Project of Hubei Cancer Hospital (Grant 2025HBCHHHRC003), the Health Commission of Hubei Province Scientific Research Project (Grant WJ2023Z011), and the Traditional Chinese Medicine Research Project of the Hubei Provincial Health Commission (Grant ZY2023Z005).

## Disclosure

All authors have read and agreed to the published version of the manuscript.

## Conflicts of Interest

The authors declare no conflicts of interest.

## Data Availability

Data sharing is not applicable to this article as no datasets were generated or analyzed during the current study.
